# Factors influencing erythrocyte sedimentation rate in adults: New evidence for an old test

**DOI:** 10.1097/MD.0000000000016816

**Published:** 2019-08-23

**Authors:** Vanessa Alende-Castro, Manuela Alonso-Sampedro, Nuria Vazquez-Temprano, Carmen Tuñez, Daniel Rey, Carmen García-Iglesias, Bernardo Sopeña, Francisco Gude, Arturo Gonzalez-Quintela

**Affiliations:** a Department of Internal Medicine, Complejo Hospitalario Universitario, University of Santiago de Compostela; b Department of Clinical Epidemiology, Complejo Hospitalario Universitario, Santiago de Compostela; c Primary Care Center, A Estrada, Spain.

**Keywords:** age, alcohol, erythrocyte sedimentation rate, gender, metabolic syndrome, obesity, physical exercise, smoking

## Abstract

The erythrocyte sedimentation rate (ESR) is a routine test for inflammation. Few studies have investigated the potential influence of lifestyle factors and common metabolic abnormalities on the ESR. This study investigates the influence of demographic factors, alcohol consumption, smoking, physical activity, obesity, and metabolic syndrome on the ESR in adults.

This cross-sectional study covered 1472 individuals (44.5% males; age range, 18–91 years) randomly selected from the population of a Spanish municipality. The ESR was measured using a standardized method. We assessed habitual alcohol consumption in standard drinking units, along with tobacco smoking, regular physical exercise (by questionnaire), body mass index, and variables defining metabolic syndrome. Multivariate analyses were performed, including mean corpuscular volume and hemoglobin concentration in the models.

The ESR was higher in females than in males, and increased steadily with age. Median ESR of females was 2-fold higher than that of males, and median ESR of individuals aged >65 years was 2-fold higher than that of individuals in the youngest category (ages 18–35 years). Body mass index, presence of metabolic syndrome, and smoking were independently and positively associated with higher ESR values. Light alcohol drinkers and individuals with high regular physical activity displayed lower ESR values than did alcohol abstainers and individuals with low physical activity, respectively.

ESR varies greatly with age and sex, and corresponding reference values are proposed. Lifestyle factors (physical activity, smoking, and alcohol consumption) and common metabolic abnormalities (obesity and related metabolic syndrome) may also influence ESR values.

## 1 Introduction

The erythrocyte sedimentation rate (ESR) is an inflammation marker used in routine clinical practice. Although it was 1st described more than a century ago,^[1]^ its clinical utility remains unaltered.^[2]^ The ESR measures the rate (mm/h) at which red blood cells form aggregates (or *rouleaux*) that sediment when anticoagulated fresh blood is left in a vertical tube.^[3]^ It is therefore not the measure of an analyte but of a physical phenomenon.^[3–5]^ It has been suggested that the term “ESR” should be changed to “length of sedimentation reaction in blood,”^[4,6,7]^ though this denomination is not widely accepted. The ESR is an estimator of overall inflammation because it depends on the concentration of acute-phase proteins circulating in the blood, particularly fibrinogen; these proteins increase the dielectric constant in the blood and neutralize the negative charges on the surface of red blood cells, which repel one another and physiologically oppose aggregation.^[3,5]^ Despite its limitations and the introduction of more specific inflammation markers, the ESR is still widely used for diagnosis and monitoring of a variety of conditions, particularly infections and rheumatic diseases.^[2]^ ESR sensitivity and specificity are not high but the test has the advantages of familiarity, simplicity, speed, low cost, and extensive coverage in the literature.^[4,5,7,8]^

Determining the ESR in general populations is important for interpreting reference values. The guidelines for definition and determination of reference intervals indicate that partitioning should be considered when there are significant differences among subgroups defined by age, sex, and common exposures.^[9,10]^ The ESR increases with age in adults,^[5,6,11–14]^ and at a given age is higher in females than in males.^[5,6,12–14]^ Common metabolic abnormalities, such as obesity and the related metabolic syndrome, are proinflammatory states which can be associated with increased ESR.^[15–17]^ With regard to lifestyle factors, previous studies have reported lower ESRs in relation with physical activity in a selected elderly population.^[18]^ Previous studies have also shown that smoking increases ESR in females^[19]^ and in selected samples of patients with arthritis.^[20]^ The potential effect of additional lifestyle factors, such as alcohol consumption, on ESR has been not fully explored. Heavy drinking has proinflammatory effects; in fact, alcoholic liver disease is a paradigm of inflammatory disorder.^[21]^ Hence, the ESR is increased in patients with complications of alcohol abuse^[22]^ and in those with alcoholic hepatitis.^[21]^ However, moderate alcohol consumption is associated with lower ESR values in selected samples of patients with inflammatory diseases, both high grade (chronic arthritis)^[20]^ and low grade (coronary disease).^[23]^ Lastly, the ESR may be influenced by the number, volume, and shape of erythrocytes, tending to be higher in patients with anemia or high red blood cell volume.^[2,5,24]^

To the best of our knowledge, there are no studies that have comprehensively assessed the potential effect of demographic (age and sex), metabolic (obesity and metabolic syndrome), and lifestyle factors (alcohol consumption, smoking, and physical activity) on ESR in a general adult population. The present study sought to investigate the potential effect of these factors on ESR. The results were further adjusted for mean corpuscular volume (MCV) and hemoglobin concentration. An approach to determining ESR reference values was developed.

## 2 Methods

### 2.1 Design

**Figure 1:**
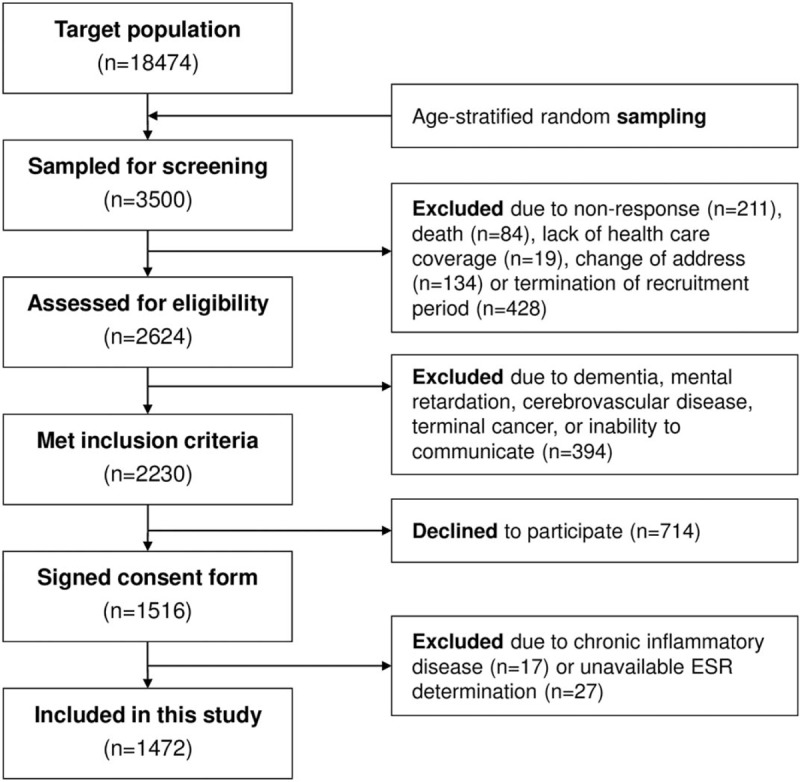
Flowchart showing study profile. ESR = erythrocyte sedimentation rate.

We conducted a cross-sectional study in the municipality of A-Estrada (Northwestern Spain, location 42°41′21″N, 8°29′14″W). An outline of the study (AEGIS, A-Estrada Glycation and Inflammation Study) is available at www.clinicaltrials.gov, code NCT01796184. The municipality had an adult population (age >18 years) of 18,474 when the study started in 2012. A flowchart of the study profile is shown in Figure 1. An age-stratified random sample of the population aged 18 years and older was drawn from Spain's National Health System Registry, which covers more than 95% of the population and contains the name, date of birth, and address of every person entitled to primary care. The sample was stratified into the following age groups: 18 to 29 years; 30 to 39 years; 40 to 49 years; 50 to 59 years; 60 to 69 years; 70 to 79 years; and 80 years and older. A computer program generated a random sample of an equal number (n = 500) of subjects in each age group. Of this initial sample of 3500 individuals, 2230 could be assessed for eligibility and displayed no exclusion criteria (Fig. 1); of these, 1516 individuals agreed to participate (overall participation rate, 68%). Participation was lower in males than in females (65% vs 71%). There were no significant differences in terms of age or residence (rural vs urban) between participants and nonparticipants. From November 2012 through March 2015, all subjects were successively contacted and asked to attend the Primary Care Center for evaluation, which included an interviewer-administered structured questionnaire and fasting venous blood sampling. The ESR was unavailable for technical reasons in 27 individuals. In addition, 17 individuals were excluded from this specific study, due to chronic inflammatory diseases that could influence the ESR. The final study population thus included 1472 individuals, 655 (45%) of whom were males. Median age was 52 years (range, 18–91 years).

### 2.2 Ethical issues

Written informed consent was obtained from all participants. The study was approved by the Regional Ethics Committee (code 2010-315) and conformed to the current Helsinki Declaration.

### 2.3 Assessment of alcohol consumption

Alcohol consumption was evaluated in standard drinking units,^[25]^ by summing the number of glasses of wine (∼10 g), bottles of beer (∼10 g), and units of spirits (∼20 g) regularly consumed per week. Individuals with an alcohol consumption of 1 to 140 g/wk were defined as light drinkers, those with an alcohol consumption of 141 to 279 g/wk were defined as moderate drinkers, and those with an alcohol consumption ≥280 g/wk were defined as heavy drinkers. Median alcohol consumption in heavy drinkers was 350 g/wk (range, 280–1100 g/wk). The remainder, comprising alcohol abstainers and occasional alcohol drinkers, were included in the same group.

### 2.4 Assessment of smoking

Consumers of at least 1 cigarette per day were deemed to be smokers. Individuals who had quit smoking during the preceding year were still considered smokers, while those who had quit more than 1 year prior to the study were considered ex-smokers.

#### 2.4.1 Definition of metabolic abnormalities

Body mass index (BMI) was calculated as weight (in kilogram) divided by the square of height (in meters). Following standard criteria, individuals were classified as normal weight (<25 kg/m^2^), overweight (25–30 kg/m^2^), or obese (>30 kg/m^2^).

Metabolic syndrome was defined according to Adult Treatment Panel III criteria,^[26]^ namely: abdominal obesity (waist circumference >102 cm in males or >88 cm in females); hypertriglyceridemia (fasting serum triglycerides ≥150 mg/dL); low high-density lipoprotein (HDL)-cholesterol levels (fasting HDL-cholesterol <40 mg/dL in males or <50 mg/dL in females); increased blood pressure (arterial blood pressure ≥130/≥85 mm Hg or current antihypertensive medication use); and hyperglycemia (fasting serum glucose ≥110 mg/dL or current antidiabetic therapy). Individuals who met at least 3 of these criteria were classified as having metabolic syndrome.

### 2.5 Assessment of physical activity

All study participants completed the International Physical Activity Questionnaire (short version). The questionnaire is freely available at https://sites.google.com/site/theipaq/home and has been validated in Spain.^[27]^ The questionnaire allows for the calculation of Metabolic Equivalents of Task (MET) and for stratification of habitual physical activity as low, moderate, or high.^[28]^ Individuals were considered to display moderate physical activity when they met any one of the following 3 criteria: 3 or more days of vigorous activity of at least 20 minutes per day or 5 or more days of moderate-intensity activity or walking of at least 30 minutes per day or 5 or more days of any combination of walking, moderate-intensity or vigorous intensity activities achieving a minimum of at least 600 MET-min/wk. Individuals were considered to display high physical activity when they met any one of the following 2 criteria: vigorous-intensity activity on at least 3 days and accumulating at least 1500 MET-min/wk, or 7 days of any combination of walking, moderate-intensity or vigorous intensity activities achieving a minimum of at least 3000 MET-min/wk. Those individuals who did not meet criteria for moderate or high physical activity were considered low/inactive.

### 2.6 Erythrocyte sedimentation rate assay

The ESR was measured in an automated TEST-1 device (Alifax, Padua, Italy), which uses microsedimentation and quantitative capillary photometry technology. The system uses an infrared ray microphotometer with a light wave length of 950 nm. The electrical impulses are collected by a photodiode detector and are correlated to the concentration of red blood cells at that capillary level. The impulses measured per unit of time are then used to delineate a sedimentation curve for each sample.^[7]^ The test was designed to overcome some drawbacks of the Westergren method; it allows for calculation of ESR in low blood volumes (150 μL) and detects the formation of *rouleaux* in 20 seconds. Determination of ESR by this method appears to be less affected by hemoglobin and corpuscular volume.^[6,29]^ The TEST-1 has been validated in relation to the reference Westergren method^[4,6]^ following the International Council for Standardization in Hematology criteria.^[2]^ Similar devices are currently used for ESR determination in most clinical laboratories around the world.^[2]^ According to the manufacturer's instructions, the coefficient of variation is 4.8% for samples with normal ESR and 5.0% for samples with high ESR. For this study, blood was drawn in vacuum tubes containing K_3_EDTA (Becton Dickinson, Franklin Lakes, NJ). Determination of ESR was performed within the following 4 hours. The same blood sample was used for measuring hemoglobin concentration and MCV. The method, devices, and personnel for ESR determination were the same as those routinely used at our hospital for clinical purposes. The hospital's reference ESR values are 0 to 20 mm/h for males and 0 to 30 mm/h for females.

### 2.7 Statistical analyses

The Mann–Whitney test was used to compare numerical variables. Different distributions were fitted using Generalized Additive Models for Location Scale and Shape (GAMLSS).^[30]^ GAMLSS provide a flexible modeling framework for responses from a large class of distributions, including the normal distribution, as well as highly skewed and kurtotic continuous distributions. GAMLSS allows for the modeling, not only of the mean response μ (i.e., location), but also of other distribution parameters, such as standard deviation σ (i.e., scale) or skewness and kurtosis (i.e., shape parameters), as a function of a set of explanatory variables. The following GAMLSS formulation was used for modeling their corresponding mean and scale parameters:  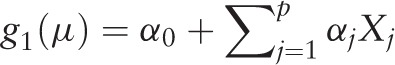



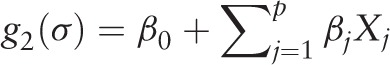


where *X*_*j*_ (*j* = 1,...,*p*) are the set of *p* covariates and *g*_*k*_ (*k* *=* 1,2) are monotonic link functions relating the parameters *μ* and *σ* to the covariates. The influence of age and all other continuous covariates on parameters of the distributions considered were modeled, either as a constant, a linear function, or a penalized spline of the covariate.^[31]^ Goodness-of-fit was assessed by the Bayesian Information Criterion and Q-Q plots, to select best fitting distribution model (Generalized Inverse Gaussian [GIG], which is appropriate for highly positive skewed data) and the influence of covariates on the distribution parameters. Worm plots were used as a diagnostic tool to assess whether adjustment for kurtosis and/or skewness was required.^[32]^ Percentile curves of ESR as a function of covariates (age and sex) were calculated on the basis of the GAMLSS regression models that displayed the best goodness-of-fit. To facilitate the clinical use of percentile curve data, we defined cut points for ESR at the 90th, 95th, and 97.5th percentiles. All statistical analyses were performed using the R statistical software environment (version 3.0.2; R Foundation, http://www.r-project.org) with the “gamlss” package.^[33]^

## 3 Results

### 3.1 Descriptive results and influence of demographics on the ESR

**Figure 2:**
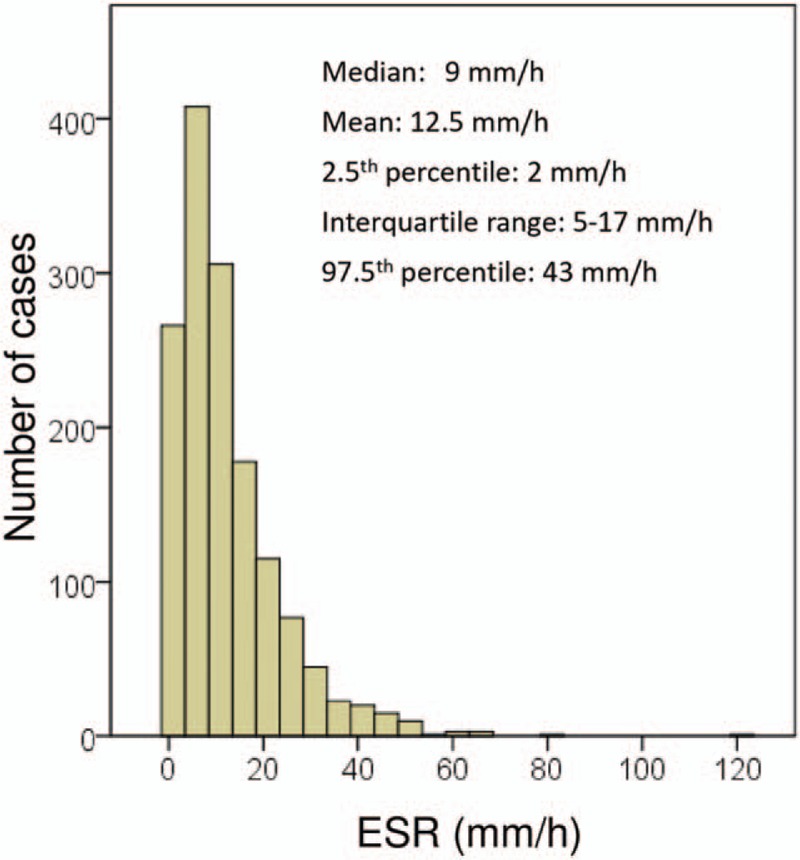
Histogram of erythrocyte sedimentation rate (ESR) distribution in the study population.

**Figure 3:**
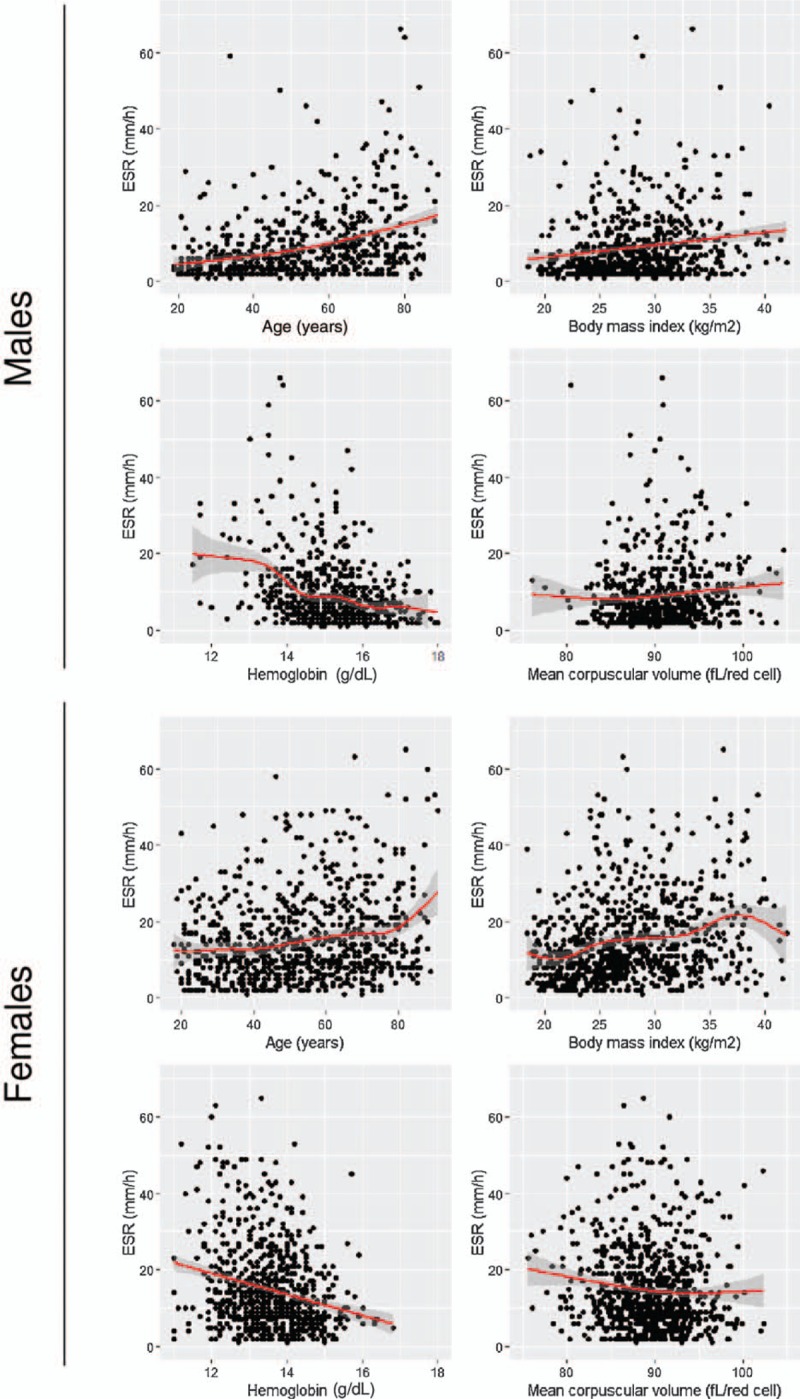
Relationship between erythrocyte sedimentation rate (ESR) and age, body mass index, blood hemoglobin concentration, and red blood cell mean corpuscular volume in males and females.

Overall, ESR values for the study population as a whole ranged from 1 to 120 mm/h (median value, 9 mm/h). A histogram of ESR results is depicted in Figure 2. The ESR was significantly higher in females (median 12 mm/h and interquartile range 7–21 mm/h) than in males (median 6 mm/h and interquartile range 3–12 mm/h; *P* < .001). For that reason, the ESR values in males and females are shown separately (Table 1). The ESR significantly increased with age in both males and females (Table 1). The association between ESR and sex and age was maintained after adjustment for additional covariates, including alcohol consumption, smoking, physical activity, BMI, metabolic syndrome, hemoglobin concentration, and MCV (Table 2). Moreover, age and sex were the strongest factors associated with ESR in that model, along with blood hemoglobin concentration (Table 2). The relation between age, hemoglobin, MCV, and ESR in males and females is further depicted in Figure 3.

**Table 1:**
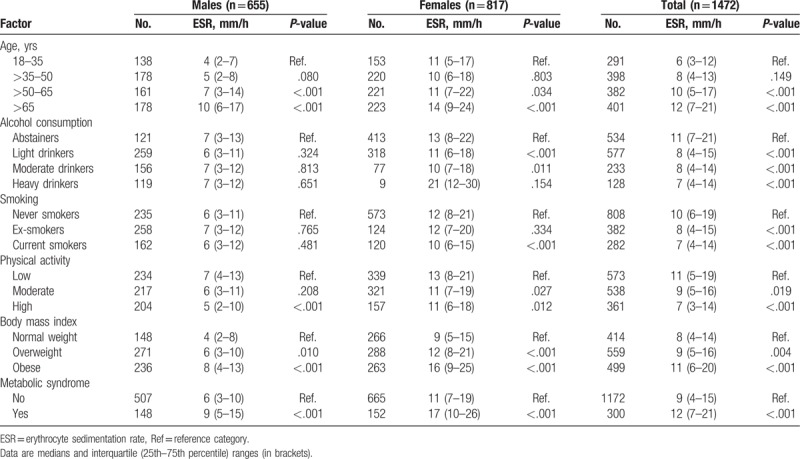
ESR in relation to age, lifestyle factors, and metabolic abnormalities in males and females.

**Table 2:**
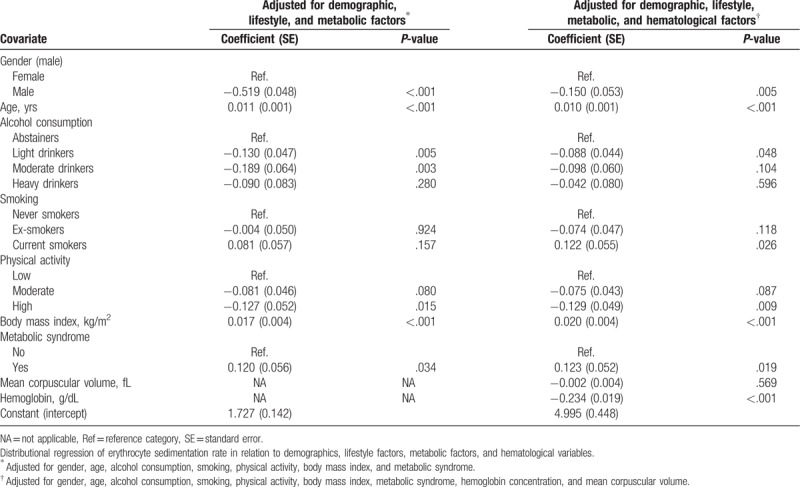
Multivariate analyses.

A total of 141 individuals (9.6%) registered ESRs higher than the laboratory threshold for normality in males and females (20 and 30 mm/h, respectively). Suggested ESR reference values based on the average predicted value for different ages in males and females are shown in Table 3.

**Table 3:**
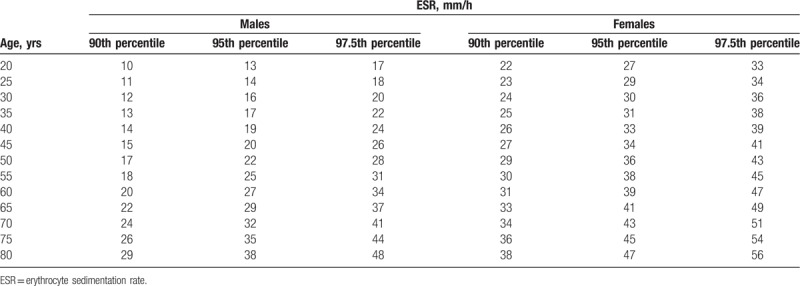
Suggested ESR reference points based on the average predicted value within each age-and-sex group, showing the 90th, 95th, and 97.5th percentiles in each category.

### 3.2 Influence of alcohol consumption on the ESR

Alcohol consumption was negatively associated with the ESR, that is, regular drinkers of low, moderate, and high quantities of alcohol registered a lower ESR than did abstainers/occasional drinkers in the univariate analysis (Table 1). This negative association was particularly evident among females (Table 1). The relationship between light and moderate alcohol consumption and a lower ESR was still present after adjustment for age, sex, smoking, physical activity, BMI, and metabolic syndrome (Table 2). However, the association became attenuated after adjusting for hematologic covariates (hemoglobin concentration and MCV), with the result that only the association between light alcohol consumption and lower ESR values remained statistically significant (Table 2).

### 3.3 Influence of smoking on the ESR

Smokers displayed a lower ESR than did never smokers in the univariate analysis, particularly among females (Table 1). However, this association became attenuated and even tended to become inverse after adjusting for covariates (Table 2). In these models, current smokers registered higher ESR values than did never smokers.

### 3.4 Influence of physical activity on the ESR

Moderate and high regular physical exercises were negatively associated with ESR in the univariate analysis (Table 1). High regular physical exercise was associated with lower ESR after adjustment for covariates (Table 2).

### 3.5 Influence of metabolic factors on the ESR

Overweight, obesity, and the metabolic syndrome were positively associated with ESR in both males and females (Table 1). The association between BMI and metabolic syndrome, and higher ESR values was still present after adjusting for covariates (Table 2). The relationship between BMI and ESR in males and females is further depicted in Figure 3.

## 4 Discussion

The results of our study show that ESR values in a general adult population are strongly influenced by age and sex. Moreover, lifestyle variables (regular physical exercise, alcohol consumption, and smoking) and common metabolic abnormalities (metabolic syndrome and high BMI) can influence ESR values in this population. Importantly, these associations remain after adjustment for covariates as well as for MCV and hemoglobin concentration, which is also a strong determinant of ESR. To the best of our knowledge, this is the 1st study to have comprehensively examined the influence of all these variables in a large general adult population sample.

The effect of age and gender on ESR has previously been described. It is known that the ESR increases with age^[5,6,11–14]^ and is higher in females than in males.^[5,6,12–14]^ According to our results, these differences are large enough to propose different reference values. Simplistic approaches which only establish an average reference value for males and females may therefore be inaccurate.

The effect of alcohol consumption on ESR has not been fully investigated to date. Anecdotally, Sykes^[34]^ reported that contamination of blood with small ethanol doses induced lower ESR. Bain concluded that alcohol consumption had no significant influence on the ESR but his sample was highly selected (386 healthy workers from a hospital staff aged 18–59 years); moreover, classification of alcohol consumption was very simplistic^[12]^ and therefore nondifferential misclassification could have biased the results to the null. More recent studies have mentioned alcohol consumption but have not analyzed its relationship with ESR in depth.^[13]^ In our experience, light and moderate alcohol consumption is associated with lower ESR. In univariate analyses, this association was only evident among females. In multivariate analyses, light and moderate alcohol consumption was associated with lower ESR after adjustment for age, sex, smoking, physical activity, BMI, and metabolic syndrome. However, the association became largely attenuated after adjustment for hemoglobin concentration and MCV, suggesting that alcohol-induced hematological alterations partly mediate the apparent effects of alcohol on ESR. Light alcohol consumption remained significantly associated with lower ESR values, a finding that is in agreement with studies in selected populations of patients with specific inflammatory diseases.^[22,23]^ Furthermore, these results would be in agreement with studies showing that light-to-moderate alcohol drinking has antiinflammatory effects, as revealed by lower serum concentrations of C-reactive protein (CRP).^[35,36]^ These antiinflammatory effects could underlie part of the well-known benefit of low-to-moderate alcohol consumption on overall mortality.^[37,38]^ Our study failed to find any significant association between heavy (≥280 g/wk) alcohol drinking and ESR. Previous studies have reported increased ESR in patients with complications of alcohol abuse.^[21,22]^ It should be noted that median alcohol intake among heavy drinkers in our study was 50 g/d, and median intake in a series of 138 alcoholics admitted to the hospital in the same area was 120 g/wk.^[39]^ These alcoholic patients had elevated inflammation markers,^[39]^ including ESR, which was abnormally high in 59% of them (median ESR 27 mm/h, range 8–88 mm/h, unpublished observation). Taken together, these results are consistent with an antiinflammatory effect of light-to-moderate alcohol consumption and a proinflammatory effect of excessive alcohol consumption. A similar J- or U-shaped relationship between alcohol consumption and inflammatory markers has been described for CRP and tumor necrosis factor-alpha^[35,36]^ but has not been described for the ESR. Intriguingly, the relationship between the quantity of alcohol consumption and mortality is also J-shaped.^[37,38]^

The potential effect of smoking on ESR is of interest. The univariate analyses showed that smoking is associated with lower ESR values, particularly among females. However, given that smoking is associated with male gender, alcohol consumption, and age, adjustment for these variables was followed by a change in the direction of association, that is, smoking was found to be associated with higher ESR values in the multivariate analyses. Previous studies have also shown that smoking increases ESR in selected samples of females^[19]^ and patients with arthritis.^[20,40]^

Regular physical exercise, particularly of a high level, was associated with lower ESR values, as compared to individuals with low physical activity. Likewise, previous studies reported lower ERS in relation with physical activity in a selected sample of elderly people.^[18]^ Furthermore, physical activity was observed to be negatively correlated with acute phase reactants such as CRP,^[41]^ a finding that is in agreement with an overall antiinflammatory effect of physical exercise.^[42]^ The relationship between regular exercise and ESR values in the general population has not been previously investigated. Prospective studies are required to confirm this association.

The univariate analyses showed that the ESR was higher in individuals with metabolic abnormalities, including overweight, obesity, and metabolic syndrome. In the multivariate analyses, BMI and the presence of metabolic syndrome maintained the association with higher ESR values. This finding has already been reported and is consistent with a proinflammatory state in these disorders.^[15–17]^

The study has some limitations that warrant mention. The cross-sectional design has inherent temporal ambiguity, which limits any inference of causality. The study participation rate was adequate, and there is no reason to suspect that selection might have biased the results; indeed, the fact that the study was population based and that the participants were randomly selected can be considered strengths. The authors acknowledge that confounding is a limitation of the study (as with other observational studies). Age and gender are associated with alcohol consumption, physical activity, and metabolic abnormalities. To disentangle their potential confounding effects, multivariate analyses were performed. The sample size afforded sufficient power to adjust for covariates. The ESR was determined using a commercial method that has been validated with the reference method and is widely used for clinical purposes.^[2,4,6,7,29]^ This method appears to be less affected by anemia and size of erythrocytes than is the traditional Westergren method,^[6,29]^ and the results were further adjusted for MCV and blood hemoglobin concentration.

Despite being old and unspecific, ESR remains a routine test in clinical practice. From a mechanistic stance, our findings are consistent with a proinflammatory effect of metabolic abnormalities and smoking, as well as with an antiinflammatory effect of light-to-moderate alcohol consumption and physical exercise. From a clinical standpoint, our results highlight the importance of adjusting for age, gender, alcohol consumption, smoking, physical activity, and metabolic abnormalities in studies investigating ESR-related factors in the population. Furthermore, demographic factors are strong enough to define age-and-gender reference ESR values, as proposed by this study.

## Author contributions

**Conceptualization:** Bernardo Sopeña, Francisco Gude, Arturo Gonzalez-Quintela.

**Data curation:** Vanessa Alende-Castro, Manuela Alonso-Sampedro, Nuria Vazquez-Temprano, Carmen Tuñez, Daniel Rey, Carmen García-Iglesias.

**Funding acquisition:** Francisco Gude, Arturo Gonzalez-Quintela.

**Investigation:** Vanessa Alende-Castro, Manuela Alonso-Sampedro, Carmen Tuñez, Daniel Rey, Carmen García-Iglesias.

**Methodology:** Bernardo Sopeña, Francisco Gude, Arturo Gonzalez-Quintela.

**Project administration:** Manuela Alonso-Sampedro, Arturo Gonzalez-Quintela.

**Supervision:** Francisco Gude, Arturo Gonzalez-Quintela.

**Writing – original draft:** Vanessa Alende-Castro, Nuria Vazquez-Temprano, Arturo Gonzalez-Quintela.

**Writing – review & editing:** Manuela Alonso-Sampedro, Bernardo Sopeña, Francisco Gude.

Arturo Gonzalez-Quintela orcid: 0000-0002-6909-1807.
